# News and Coming Events

**Published:** 1907-10-26

**Authors:** 


### NEWS AND COMING EVENTS.

The foundation-stone of the new extension to the Gateshead Children's Hospital was laid on the 9th inst. The designs provide for two large wards of sixteen beds each, together with operating theatre and necessary additional buildings.

The first children's sanatorium was opened recently by the Duke of Northumberland, at Stannington. The institution owes its inception to the charitable enterprise of the Poor Children's Holiday Association and Rescue Agency, and has been erected at a total cost of £14,000.

Under the will of the late Dr. Edward Capron, of Northwood Asylum, Winterbourne, Gloucestershire, the Royal Surrey County Hospital will receive nearly £14,000. Dr. Capron also bequeaths a sum of £500 to the Society for the Protection of Animals from Vivisection.

Mr. H. W. Cook contributes a series of articles on "England's First Cottage Hospital" to the *North Star*. It appears that to the North Ormsby Hospital at Middlesbrough, belongs the credit of being the first cottage hospital started in England. The hospital was opened in 1859, and originally consisted of three cottages. It was founded mainly by Sister Mary, of the Sisterhood of the Holy Rood, a Church of England institution whose headquarters were at Redcar.

In that excellent monthly, *The American Journal of Clinical Medicine*, Dr. J. M. French tells the story of the Æsculapian Club, a unique organisation consisting of five medical men and their wives, constituting a "social medical club," which appears to have had a great measure of success. "We mix just enough of the professional element with the social to prevent our forgetting that we are doctors, and that is all." There seems room here for such a club.

Mr. Justice Walton will deliver the inaugural address at the Medico-Legal Society's first meeting at 22 Albemarle Street, W., on October 29, at 8.15.

The Chelsea Hospital for Women has received from Earl Cadogan a donation of £500, and the Council has resolved to name a ward of the hospital in memory of its late benefactress, the Countess Cadogan.

The inaugural address of the Winter Session of the London School of Tropical Medicine, Connaught Road, Albert Dock, E., was delivered in the lecture theatre of the school, by Sir Lauder Brunton, M.D., LL.D., on Monday, October 21, 1907.

The Editors of the *Medical Directory* have decided to abbreviate the entries under the various societies which have lately become constituent societies of the Royal Medical Society to the following :—Fell.Roy.Soc.Med. or Mem. . . . Sect.Roy.Soc.Med. They will be glad if all Fellows of the Society, or members of sections will kindly give the necessary information to enable them to make the desired alterations in the forthcoming issue of the Directory.

It does not fall to all of us to look back on forty-six years' connection with hospital engineering. This, however, has been the experience of Mr. Thomas Cantrell, the late secretary of Messrs. Manlove Alliott and Co., Ltd., of Nottingham, well known as builders of steam disinfectors, laundry machinery, refuse destructors, and other sanitary apparatus. Failing health made it desirable that Mr. Cantrell should take a well-earned rest after nearly half a century's service with the firm mentioned, and the opportunity was taken on October 7 of presenting him with a silver tea service and salver as a recognition by the directors, staff, and workmen of his long association with them.

